# Efficacy and Safety of CAP7.1 as Second-Line Treatment for Advanced Biliary Tract Cancers: Data from a Randomised Phase II Study

**DOI:** 10.3390/cancers12113149

**Published:** 2020-10-27

**Authors:** Ulrich-Frank Pape, Stefan Kasper, Johannes Meiler, Marianne Sinn, Arndt Vogel, Lothar Müller, Oswald Burkhard, Karel Caca, Steffen Heeg, Petra Büchner-Steudel, Victor Rodriguez-Laval, Anja A Kühl, Ruza Arsenic, Holger Jansen, Peter Treasure, Nalân Utku

**Affiliations:** 1 Department of Hepatology and Gastroenterology, Campus Charité Mitte and Virchow Klinikum, Charité Universitätsmedizin Berlin, 10117 Berlin, Germany; 2 Germany and Department of Internal Medicine and Gastroenterology, Asklepios Klinik St. Georg, Asklepios Tumorzentrum, Hamburg ATZHH, 20099 Hamburg, Germany; 3 Department of Medical Oncology, West German Cancer Center, University Hospital Essen, 45147 Essen, Germany; stefan.kasper@uk-essen.de (S.K.); johannes.meiler@hancken.de (J.M.); 4 Department of Medical Oncology, Universitäts Klinikum Hamburg-Eppendorf, 20251 Hamburg, Germany; ma.sinn@uke.de; 5 Department of Gastroenterology, Campus Charité Mitte and Virchow Klinikum, Charité Universitätsmedizin Berlin, 10117 Berlin, Germany; 6 Department of Gastroenterology, Hepatology and Endocrinology, Hannover Medical School, 30625 Hannover, Germany; Vogel.Arndt@mh-hannover.de; 7 Onkologische Schwerpunktpraxis Leer-Emden-Papenburg, 26789 Leer, Germany; lothar.mueller@onkologie-ue.de; 8 Onkologische Praxis, 67547 Worms, Germany; onkopraxisworms@freenet.de; 9 Klinikum Ludwigsburg, 71640 Ludwigsburg, Germany; karel.caca@rkh-kliniken.de; 10 Department of Medicine II, Gastroenterology, Hepatology, Endocrinology and Infectious Diseases, Medical Center—University of Freiburg, Faculty of Medicine, University of Freiburg, 70085 Freiburg, Germany; steffen.heeg@uniklinik-freiburg.de; 11 Martin-Luther-University Halle Wittenberg, Medizinische Fakultät, Universitätsklinik und Poliklinik für Innere Medizin I, 06120 Halle (Saale), Germany; petra.buechner-steudel@uk-halle.de; 12 Hospital Universitario de La Princesa, 28006 Madrid, Spain; vrlaval@salud.madrid.org; 13 iPATH.Berlin, Core Unit of the Charité, Hindenburgdamm, 12203 Berlin, Germany; Anja.Kuehl@charite.de; 14 Instituts für Histologische und Zytologische Diagnostik AG, 5000 Aarau, Switzerland; ruza.arsenic@patho-diagnostik.ch; 15 Institute for Medical Immunology, Charité Universitätsmedizin Berlin, 13353 Berlin, Germany; holger.jansen@cellact.eu; 16 Peter Treasure Statistical Services Ltd., Stow Bridge PE34 3NR, UK; statistics@petertreasure.com; 17 CellAct Pharma, 44137 Dortmund, Germany

**Keywords:** biliary tract cancer, unresectable, cholangiocarcinoma, CAP7.1

## Abstract

**Simple Summary:**

Advanced biliary tract cancer is difficult to treat, and 5-year survival is less than 5% for tumours that cannot be removed by surgery. CAP7.1 is a drug being investigated for biliary tract cancer. This study assessed treatment with CAP7.1 in patients with advanced biliary tract cancer whose disease had progressed despite receiving other treatments. One group of patients received CAP7.1 together with best supportive care (BSC) and another group received BSC from their physician. The patients receiving BSC were subsequently given CAP7.1 if their disease was seen to progress. Disease control in those receiving CAP7.1 was better than that observed in patients who received BSC, with an associated greater time to disease progression. Side effects were as expected for this type of anti-cancer drug, related to dose of CAP7.1, and manageable. CAP7.1 may offer a new treatment option for biliary tract cancer and should undergo further clinical investigation.

**Abstract:**

CAP7.1 is a novel topoisomerase II inhibitor, converted to active etoposide via carboxylesterase 2 (CES2), with signals of efficacy in treatment-refractory solid tumours. In a Phase II trial, 27 patients with advanced biliary tract cancers (BTC) were randomised 1:1 to CAP7.1 plus best supportive care (BSC), or BSC alone, with crossover to CAP7.1 upon disease progression. The primary objective was disease control rate (DCR) following 28-day cycles of CAP7.1 (200/150 mg/m^2^; iv), or BSC until progression. Secondary objectives included progression-free survival (PFS), time-to-treatment failure (TTF), overall survival (OS) and safety. Fourteen patients received CAP7.1 and 13 BSC. DCR favoured CAP7.1 vs. BSC (50% vs. 20%; treatment difference: 30%, 95%CI −18.44, 69.22, full analysis set [FAS]), with disease progression in 40% vs. 70%, respectively. Significantly longer median PFS was achieved for CAP7.1 vs. BSC: 66 vs. 39 days, respectively (hazard ratio [HR] 0.31; 95%CI 0.11, 0.86; *p* = 0.009; FAS). Similar trends were observed for TTF and OS. CES2-positive patients had longer median PFS (158 vs. 56 days) and OS (228 vs. 82 days) vs. CES2-negative patients. Adverse events were predictable, dose-dependent and consistent with those previously observed with etoposide. These efficacy and safety findings in second-line BTC warrant further clinical investigation of CAP7.1.

## 1. Introduction

Biliary tract cancers (BTC) including intrahepatic, perihilar and distal cholangiocarcinoma (CCA) as well as gallbladder cancer (GBC) are rare diseases [1]. Global burden of disease estimates suggest that of the 8.9 million deaths caused by neoplasms in 2016, 161,600 were due to BTC [2]. Moreover, the incidence and mortality of BTC are increasing, making it one of the fastest rising cancers worldwide [3,4,5,6]. Prognosis for BTC is poor; where surgery is a potential curative option, 5-year survival is 33.3%, but it is only 4.1% for unresectable tumours [7,8]. Most patients present with unresectable disease and, therefore, standard of care focuses on relieving symptoms [1,9]. Chemotherapy treatment depends on performance status, and with few regimens available, there is no clear survival advantage of one regimen over another [1,9].

Etoposide, a topoisomerase II inhibitor, has demonstrated efficacy in combination with other chemotherapeutics in a variety of tumours including BTC [10,11,12,13,14,15,16,17,18]; it can, however, cause significant myelosuppression [17,19,20,21] and multi-drug resistance (MDR) resulting in either inefficacy or loss of efficacy [22,23]. The novel drug CAP7.1 (EDO-S7.1) is activated by the enzyme carboxylesterase 2 (CES), which is expressed in the liver, gallbladder and gastrointestinal tract tissues [22,24], giving rise to the hypothesis that local tumour expression may permit intra-tumoural activation of CAP7.1 and thus contribute to drug accumulation, making CAP7.1 a candidate for the treatment of BTC. Through the addition of a water-soluble ester group, CAP7.1 has been shown to overcome both the dose-dependent toxicity observed with etoposide and MDR-1 mediated resistance and substrate efflux [22,25]. In a Phase I dose-escalation study in patients with a variety of advanced treatment-refractory solid tumours, CAP7.1 demonstrated an acceptable safety profile with signals of efficacy [23]. In this study, the observed disease control rate (DCR) was 47% (8/17). As best overall tumour response, one patient achieved a partial response, 11 patients achieved stable disease, and in five patients, progressive disease was observed. One patient with stage IV GBC experienced an overall survival of 25.6 months and one patient with BTC of nonspecified location achieved a progression-free survival (PFS) of 5.4 months [23].

The aim of the current study was to expand on these findings and assess the efficacy of CAP7.1 as a second-line treatment in patients with advanced BTC who have progressed despite treatment with first-line therapy, typically gemcitabine and cisplatin.

## 2. Results

### 2.1. Patients

**Figure 1:**
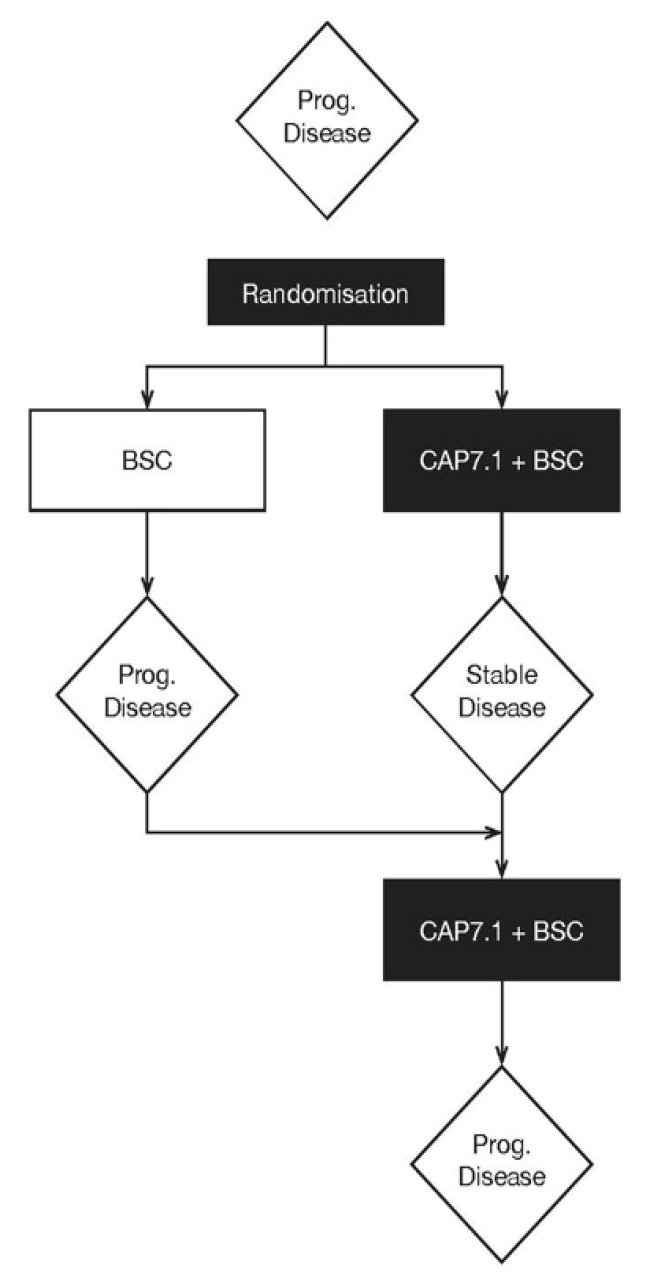
Study design for this Phase II randomised controlled trial of CAP7.1 compared with best supportive care in patients with advanced biliary tract cancer.

Twenty-seven patients with advanced BTC who had received previous standard of care therapy with gemcitabine/cisplatin were randomised: 14 to CAP7.1 and 13 to best supportive care (BSC) (Figure 1). The safety analysis set (SAS) contained 23 patients: 13 receiving CAP7.1 and 10 initially randomised to BSC who crossed over following disease progression. Three patients who were randomised to the BSC arm but were either excluded from the study at screening or withdrew consent prior to receiving any study medication did not crossover. One patient randomised to CAP-7.1 who was a late screening failure did not receive any treatment. The full analysis set (FAS) comprised 20 patients (no major eligibility violations; CAP7.1 *n* = 10, BSC *n* = 10) who received at least one dose of CAP7.1 or were treated with BSC. The per-protocol analysis set (PAS) included 19 patients from the FAS (CAP7.1 *n* = 9, BSC *n* = 10) who had no major protocol violations and appropriate follow-up assessments available for the primary endpoint evaluation of DCR (Table 1).

Demographics and baseline characteristics were generally well balanced between treatment groups (Table 2). Median age was 62.5 years. The majority of patients with information available on tumour differentiation (15/20; 75% of patients) had well or moderately differentiated BTC. Amongst those patients randomised to receive CAP7.1, 6 had extrahepatic CCA, 2 intrahepatic CCA and 1 adenocarcinoma of the ampulla of Vater. In the BSC arm of the study, 5 patients had extrahepatic CCA and 5 intrahepatic CCA.

The 10 PAS patients initially randomised to BSC all crossed over to CAP7.1 at disease progression. Overall, 14 patients (9 randomised to CAP7.1 and 5 to BSC who subsequently crossed over) received at least two cycles of CAP7.1.

### 2.2. Primary Endpoint

The proportion of patients in the FAS who achieved disease control (complete response [CR], partial response [PR], or stable disease [SD]) was numerically higher compared to BSC (Table 3): 50.0% vs. 20.0%, respectively (treatment difference: 30.0%, 95% confidence interval [CI] −18.44, 69.22; *p* = 0.175, one-sided Fisher’s exact test). A similar trend was observed in the PAS with 55.6% vs. 20.0% achieving disease control, and thus an observed treatment difference of 35.6% (95% CI −12.80, 72.39; *p* = 0.130), which was consistent with the treatment benefit of 35% anticipated in the protocol.

### 2.3. Secondary Endpoints

**Figure 2:**
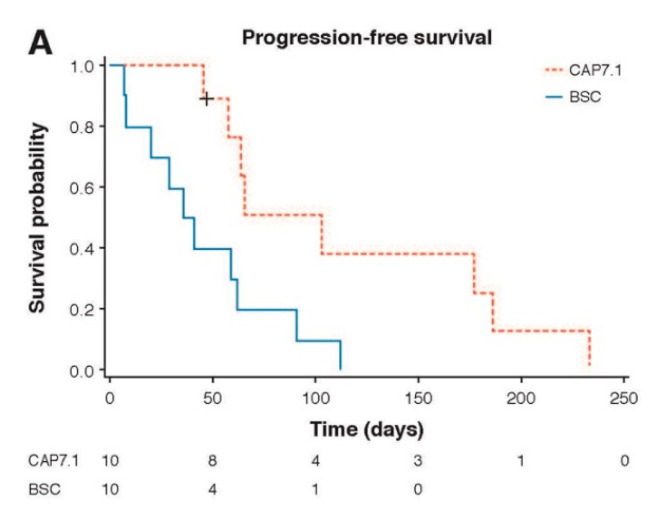

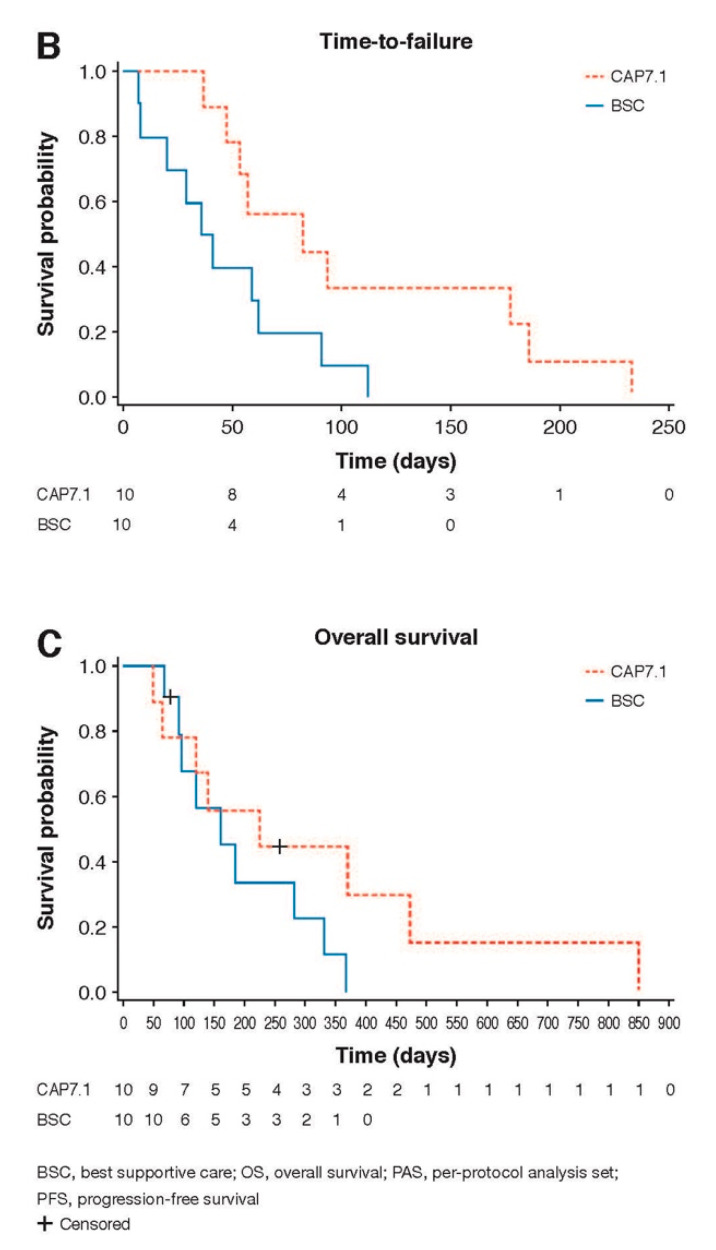
Secondary endpoint analysis results in the FAS for patients receiving early CAP7.1 vs. BSC patients who received CAP7.1 delayed after initial progression under BSC therapy: Kaplan–Meier estimates of (**A**) PFS for patients receiving CAP7.1 vs. BSC (*p* = 0.009), (**B**) TTF for patients receiving CAP7.1 vs. BSC (*p* = 0.010), and (**C**) OS for patients receiving CAP7.1 vs. BSC (*p* = 0.148).

A significantly longer median PFS was achieved for CAP7.1 compared to BSC: median 66 vs. 39 days, respectively (hazard ratio [HR] 0.31; 95%CI 0.11, 0.86; *p* = 0.009) in the FAS, and in the PAS (*p* = 0.006) (Figure 2A). Similarly, time-to-treatment failure (TTF) was statistically significantly longer for CAP7.1 compared to BSC (79 vs. 39 days, respectively), HR 0.33 (95%CI 0.12, 0.87; *p* = 0.010) in the FAS, and in the PAS (*p* = 0.006) (Figure 2B). The estimated 1-year overall survival (OS) in the FAS was 40.0% for CAP7.1 at randomisation compared to 11.3% for those who initially received BSC and were treated with CAP7.1 only after disease progression and crossover. In the FAS, median OS was 184 days with CAP7.1 vs. 162 days with BSC (Figure 2C). This trend was also supported in the PAS with a median OS from randomisation of 227 days with CAP7.1 therapy vs. 162 days with BSC (HR 0.47; *p* = 0.088).

### 2.4. Safety

The majority of patients (21/23; 91%) received at least 1 cycle of CAP7.1; 17 (74%) patients received at least 2 cycles and 5 (22%) patients received at least 5 full cycles (mean 2.6 cycles; median 2.0 cycles). Treatment duration ranged from 3 to 196 days (mean 73 days; median 56 days). Starting dose was 200 mg/m^2^ (*n* = 9), 150 mg/m^2^ (*n* = 13), and 110 mg/m^2^ (*n* = 1).

All patients in the SAS experienced at least one adverse event (AE) (Table 4). These were predictable and dose-dependent, with reversible haematological toxicity consistent to that previously observed with etoposide. No differences in the incidence of AEs was observed between the different starting doses of CAP7.1. A total of 16 serious AEs (SAEs) occurred in 7/13 (54%) patients randomised to CAP7.1; 8 of these SAEs in 6/13 (46%) patients were related to study drug. The most common drug-related AEs were leukopenia 77%, neutropenia 69%, thrombocytopenia 54%, anaemia 54%, alopecia 31%, and fatigue 31%. Grade 3–5 AEs considered to be related to the study drug and occurring in ≥5% of all patients receiving CAP7.1 were: neutropenia (57%), leukopenia (26%), anaemia, thrombocytopenia (both 17%), and febrile neutropenia, nausea, pneumonia and pyrexia (9% each). A total of 4 of the 13 patients randomised to CAP7.1 discontinued treatment due to AEs. AEs resulting in death occurred in four (31%) patients randomised to CAP7.1 with only one (tumour lysis syndrome) considered to be possibly related to the study drug. Nonrelated fatal AEs were progression of underlying disease (*n* = 2) and acute cardiac event with chest pain (*n* = 1).

### 2.5. Exploratory Efficacy Endpoints

#### 2.5.1. Post-hoc Analysis of Patients Who Crossed Over to CAP7.1

**Figure 3:**
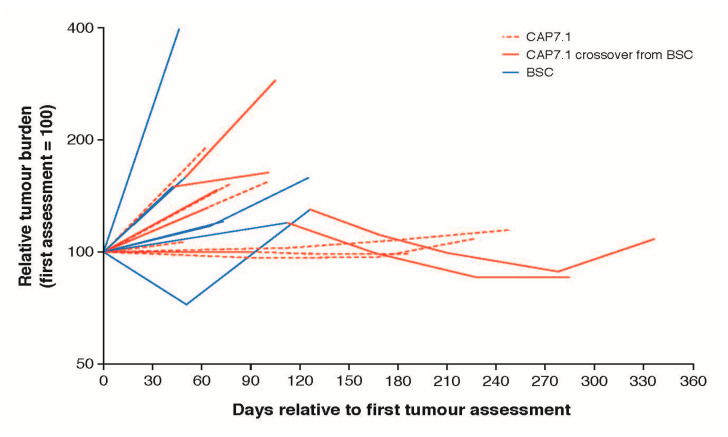
Tumour burden trajectories for 19 patients with at least 2 assessments in the FAS population.

DCR and PFS were compared before and after crossover in patients randomised to BSC. Ten patients who were eligible for inclusion in both the FAS and PAS, and who were randomised to BSC, crossed over to CAP7.1 at progression. Three of these patients (30%) who progressed on BSC achieved SD after crossover to CAP7.1, with one patient (10%) achieving a PR (total DCR 40.0% [95%CI 12.2, 73.8]) (Figure 3), compared with only two patients achieving short-term SD with BSC only (total DCR 20.0% [95%CI 2.5, 55.6]; treatment difference: 0.20 [95%CI −0.17, 0.57] *p* = 0.0786, McNemar test). The risk of progression for CAP7.1 was 2.33 times lower compared to that for BSC alone prior to crossover. In patients randomised to receive BSC, the median PFS (second progression) from crossover to CAP7.1 treatment was 50 days compared with a median PFS (first progression) whilst receiving BSC of 39 days (HR 0.43, *p* = 0.103, within-patient comparison).

**Figure 4:**
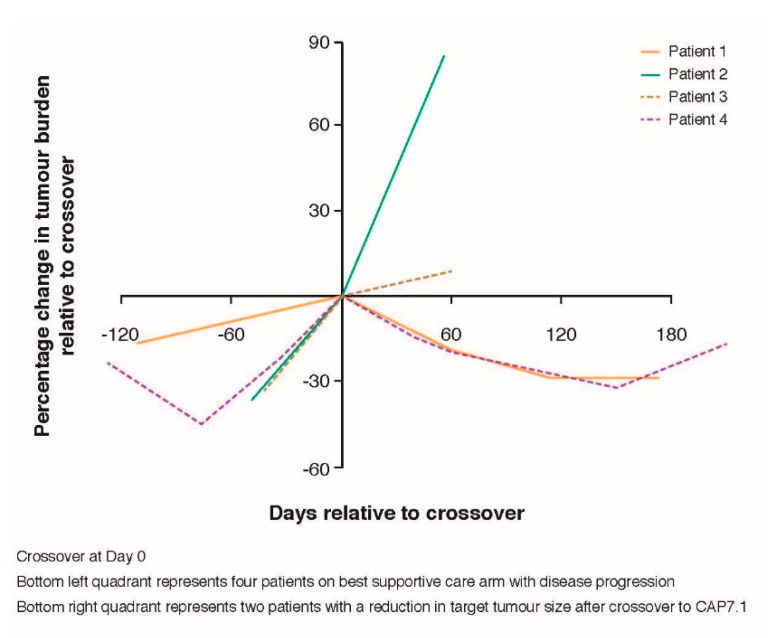
Tumour burden trajectories in four patients on best supportive care that crossed over to CAP7.1.

Following crossover to CAP7.1, a reduction in target tumour volume was observed in two patients (one with PR and one with SD), while two other patients experienced disease progression. Figure 4 shows tumour burden over time at the individual patient level for these four patients. Increased tumour burden was observed in all patients treated with BSC for whom sufficient tumour burden data were available, in contrast to the decreased or stable tumour burdens observed in those patients who received CAP7.1, with decreased tumour burden observed following crossover from BSC to CAP7.1.

**Figure 5:**
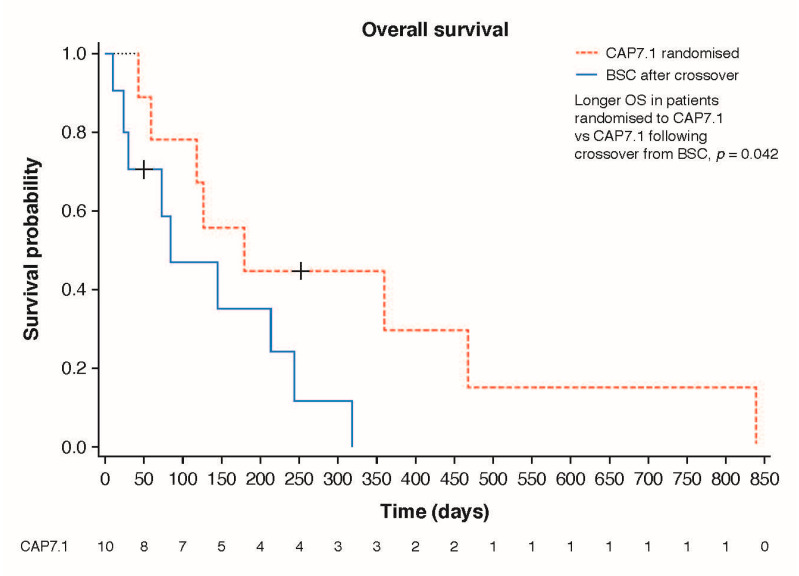

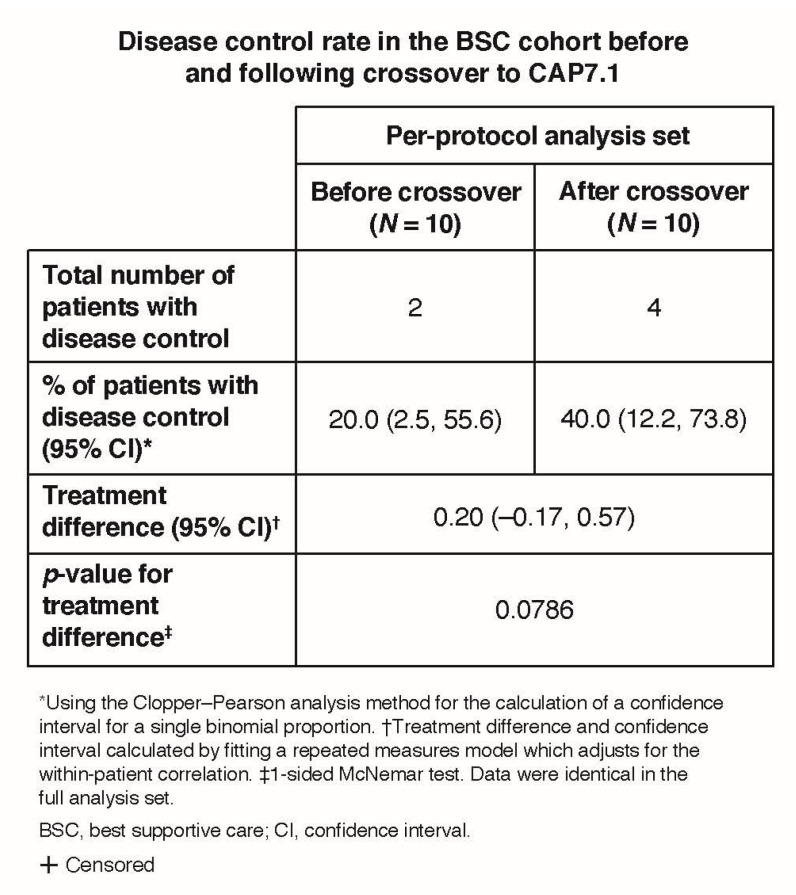
Post-hoc crossover analysis of overall survival (PAS population) and disease control rate (FAS and PAS populations) compared with patients initially randomised to CAP7.1.

Crossover from BSC to CAP7.1 was also associated with a trend for improved DCR (from 20% to 40%; *p* = 0.0786) (Figure 5). A trend for longer OS from the day of initiation of therapy was observed in patients in the FAS randomised to CAP7.1 when compared to those treated with CAP7.1 following crossover from BSC (median OS 154 vs. 83 days, respectively; HR 0.46, 95%CI 0.16, 1.30; *p* = 0.067). This between-treatment difference in OS approached significance in the PAS (median OS 180 vs. 83 days, respectively; HR 0.39; *p* = 0.042) (Figure 5). AEs were similar in those initially randomised to CAP7.1 and those who crossed over following disease progression on BSC (Table 4).

#### 2.5.2. Analysis of Tumour CES2

**Figure 6:**
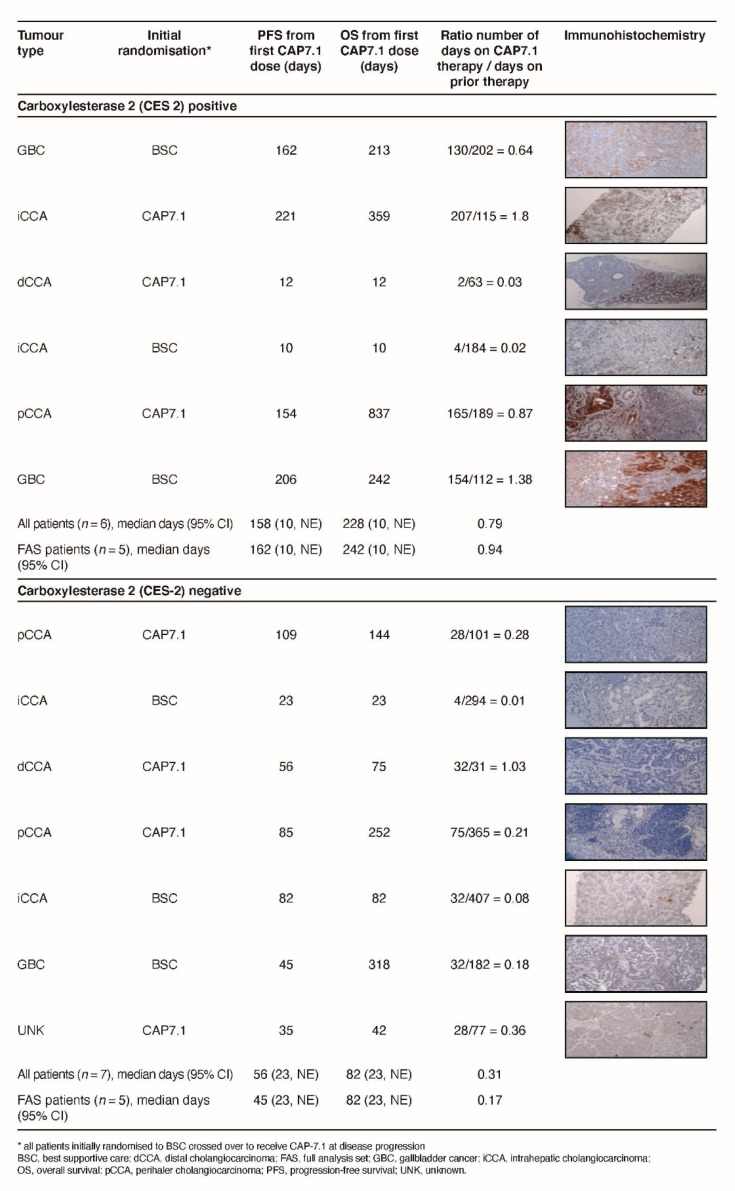
CES2 expression in tumour samples from 13 patients. (Magnification: 100×)

Tumour samples obtained from 13/27 patients were eligible for analysis of intratumour CES2 by immunohistochemistry (Figure 6). Overall, 6/13 samples were CES2-positive (CES+). CES expression was observed to be heterogeneous, and in some cases, lower than in adjacent tissues. However, in addition to expression in tumour cells, immune cell infiltrates in the immediate tumour vicinity were shown to express high levels of CES2, suggesting that in some patients, adjacent tissues may provide a relevant source of CES2. Patients with CES+ tumours demonstrated a longer median PFS (158 days [95%CI 10, not estimable (NE)] vs. 56 days [95%CI 23, NE]) and OS (228 days [95%CI 10, NE] vs. 82 days [95%CI 23, NE]) compared to those with CES2-negative (CES−) tumours. The longest observed OS of 837 days was noted in a patient with a CES+ tumour and CES+ tumour infiltrating lymphocytes. Although median OS was only 82 days in patients with CES− tumours, one such patient had an OS of 318 days.

**Figure 7:**
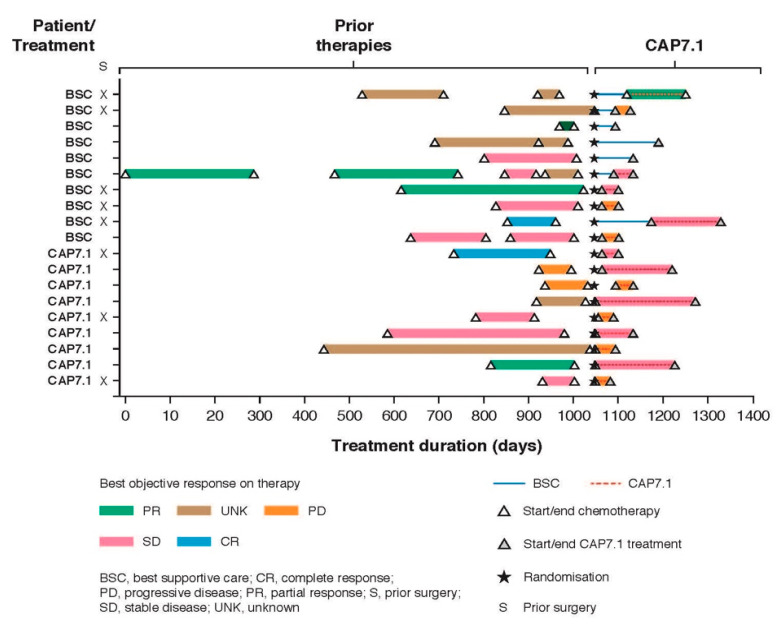
Best objective response (measured by local radiology assessment) for individual patients in the PAS population (*n* = 19) showing previous lines of chemotherapy/surgery.

The comparison of prior therapies with duration of CAP7.1 treatment showed several patients benefitted from CAP7.1 despite progression on first or subsequent lines of therapy. Figure 7 shows the best objective response (measured by local radiology assessment) as the best change in the sum of longest diameters of target lesions from the last assessment before first CAP7.1 (CAP7.1 phase) or screening (BSC phase). Five out of 19 patients in the PAS population either benefitted for longer (*n* = 2) or to a similar extent (*n* = 3) from CAP7.1 therapy in comparison to first-line treatment.

Duration of prior therapy in patients with CES+ tumours ranged from 16.0 to 33.1 weeks, and duration of CAP7.1 therapy was 18.7 to 29.7 weeks. The mean ratio of treatment duration for CAP7.1 vs. prior therapy was 1.16 (range 0.56–1.79). In comparison, although patients with CES− tumours demonstrated a duration of prior therapy of 11.0–58.1 weeks, they received CAP7.1 for a shorter duration (4.1–4.7 weeks). Therefore, the mean ratio of treatment duration for CAP7.1 vs. prior therapy in patients with CES− tumours was only 0.21 (range 0.08–0.38).

## 3. Discussion

This study demonstrated that a greater proportion of patients with BTC randomised directly to CAP7.1 achieved better disease control compared to patients randomised first to BSC who subsequently received treatment with CAP7.1. Statistical significance was not reached for the primary endpoint, most likely due to the small sample size; however, treatment with CAP7.1 resulted in greater median PFS and TTF vs. BSC. Moreover, longer 1-year OS was observed for patients randomised to CAP7.1 compared to those who crossed over after progression with BSC. The higher risk of progression with BSC prior to crossover provides further evidence for the advantage of switching from BSC to CAP7.1 treatment. Within-patient analysis of BSC patients who crossed over to CAP7.1 showed improvement in DCR and PFS following crossover, with one patient showing a partial response.

Adverse events were predictable and dose-dependent, with reversible haematological toxicity consistent to that observed for etoposide at lower doses. No dose-limiting organ toxicity was observed, despite the administration of higher doses than typically used for conventional etoposide [19,21]. These findings support the predicted tolerability profile of CAP7.1 given its conversion to an active form following administration, which allows higher dosing than with conventional etoposide.

Exploratory immunohistochemistry was undertaken to assess how CES2 tumour expression might affect CAP7.1 efficacy, given its role in the conversion of CAP7.1 to etoposide. The preliminary results obtained in a small number of patients within this study suggest a possible correlation between CES expression and patient outcomes in terms of improved PFS and OS. Further studies in a larger patient population would be required to further investigate this hypothesis.

There is a high level of unmet medical need in the treatment of advanced BTC due to both a paucity of studies investigating salvage therapy and the disappointing findings of such studies [26]. This lack of successful research has resulted in limited treatment options for patients with advanced BTC [27,28]. At the time of the initiation of this study, there was no established second-line therapy standard for BTC [1]. However, the Phase III ABC-06 trial results suggest a 1-month median OS benefit vs. active symptom control (ASC) for the modified FOLFOX plus ASC regimen in second-line BTC (6.2 months vs. 5.3 months, respectively) [29]. This study further reported an OS rate of 50.6% at 6 months and 25.9% at 12 months with modified FOLFOX + ASC compared with 35.5% and 11.4%, respectively, with ASC alone [25]. The findings from our study support the further clinical investigation of CAP7.1 in order to assess its potential to improve outcomes in patients with BTC where there are limited data and approved salvage therapies. Interestingly, numerically greater OS was observed in our study than in the ABC-06 study; however, it should be noted that these data are not comparative and that this Phase II study enrolled a significantly smaller patient population. Nevertheless, this trend provides support for the further clinical development of CAP7.1.

Strengths of this Phase II study include the relatively homogeneous late-stage patient population with metastatic BTC compared to other studies, which included a generally more diverse set of patients [11,29]. The crossover aspect of this study, whereby BTC patients could receive CAP7.1 at progression, also permitted the unique opportunity of a within-patient treatment comparison, which is independent of prognostic factor profile, a major potential confounding factor of treatment outcome. Study limitations included the small number of patients, which is in part a consequence of the rare nature of BTC, termination of the study at the interim analysis for reasons explained in the ‘Materials and Methods’, the heterogeneity in dosing of CAP7.1 and that the analysis was not conducted according to the intention-to-treat (ITT) principle. In addition, the open-label study design, with its permitted crossover from BSC to CAP7.1 following disease progression, may be regarded as a limitation of the study owing to the risk of assessment bias when determining disease progression in those patients initially randomised to BSC.

## 4. Materials and Methods

### 4.1. Study Design

This was a Phase II, multicentre, comparative, open-label study (EudraCT: 2012-002378-30; NCT02094560) to assess the anti-tumour activity of CAP7.1 in patients with advanced, relapsed/refractory BTC who had received one previous line of therapy. Patients were randomised 1:1 to receive either CAP7.1 in combination with BSC as per the institution’s standard, or BSC alone. Patients randomised to BSC alone were allowed to crossover to CAP7.1 treatment upon disease progression (Figure 1). The null hypothesis for this study was that the DCR in the CAP7.1 arm was equal to or lower than the DCR in the BSC arm. The alternative hypothesis was that the DCR in the CAP7.1 arm was higher than the DCR in the BSC arm. The original sample size was planned to be 50 patients with advanced BTC based on a group sequential design. The study was terminated at the first of two planned interim analyses, which were to be performed after 18 and 34 patients, respectively, had been randomised with stopping rules calculated using the O’Brien-Fleming method, with the overall study *p*-value preserved at 2.5% one-sided [30]. The objective of the first interim analysis was to assess the safety and efficacy of CAP7.1. Study termination occurred following discussions with the European Medicines Agency, to allow for a confirmatory regulatory study to be initiated. The results presented in this manuscript include data collected up to this point.

### 4.2. Dosing

CAP7.1 was administered via a 60 min intravenous (iv) infusion for 5 consecutive days at either 150 or 200 mg/m^2^/day (investigator’s discretion given that the maximum tolerated dose defined in Phase I was 200 mg/m^2^/day with the majority of stable responses observed at that dose level). Treatment was administered every 28 days, with cycles repeated until progression, unmanageable toxicity or withdrawal of consent (whichever occurred first), provided complete recovery from toxicities. For patients whose starting dose was 150 mg/m^2^/day, the dose could be increased to 200 mg/m^2^/day if the first two doses were well tolerated. In case of toxicities observed at 150 mg/m^2^/day, the dose could be reduced to 110 mg/m^2^/day. Patients who experienced unacceptable toxicity mid-cycle while on the 200 mg/m^2^/day dose were allowed to resume therapy at a reduced dose of 150 mg/m^2^ after recovery to Grade 1 severity or better.

### 4.3. Patients

Patients ≥18 years with confirmed diagnosis of advanced BTC, an Eastern Cooperative Oncology Group (ECOG) Performance Status of 0–2, anticipated life expectancy ≥8 weeks, adequate bone marrow function, recovery from previous myelosuppressive chemotherapy and disease progression following one chemotherapy line were included. Exclusion criteria were: any condition affecting compliance with the protocol; pregnancy or breastfeeding; infection; other cancer therapy; or participation in another clinical trial within 30 days preceding the screening visit. The study was approved for conduct by the Independent Ethics Committee Ethik-Komission II, with the study protocol, informed consent form and other relevant study-related documents submitted under Submission File No 2012-040F-MA. Patient informed consent was obtained in all patients according to GCP standards and in accordance with the Declaration of Helsinki. Ethics committee votes were obtained at each participating centre.

### 4.4. Study Endpoints

The primary efficacy endpoint was DCR, defined as the proportion of patients who achieved an objective response (CR or PR) or SD according to RECIST v1.1. Treatment difference and one-sided *p*-value for the treatment difference were also presented. Secondary study objectives included PFS, TTF (defined as time to the earliest of death, disease progression or study withdrawal), OS and safety. The efficacy analyses were performed for the FAS (patients with confirmed BTC, meeting all major eligibility criteria and, as appropriate, receiving at least one dose of CAP7.1 or BSC) and for the PAS (all patients in the FAS without major protocol violations).

Exploratory endpoints included a post-hoc analysis of patients who crossed over from BSC to CAP7.1 to determine DCR, PFS and OS, as well as an assessment of intratumour CES2 expression by immunohistochemistry. The analysis of tissue samples obtained from patients for CES status has been described elsewhere [31].

### 4.5. Measurements of Efficacy

Tumour sites were documented according to RECIST 1.1 at baseline and after every two treatment cycles of CAP7.1. For patients who achieved CR, PR or SD, an additional confirmatory assessment was performed after ≥4 weeks with scans repeated at 4- to 6-week intervals thereafter. All scans were subjected to a blinded external radiologist review. OS was a prespecified secondary endpoint for the study, with all patients followed for 6 months or until half the patients in the study had died, whichever occurred earlier. As all BSC patients eventually progressed and crossed over to CAP7.1, a meaningful comparison of OS between the two treatment groups was not possible. However, PFS was considered to be an appropriate surrogate endpoint for OS in Phase II trials in advanced BTC [32]. Analysis of tumour CES2 expression using immunohistochemistry was performed [31].

### 4.6. Measurements of Safety

AEs were recorded according to MedDRA v16.0E using standard laboratory safety assessments at baseline and weekly for the SAS. ECOG Performance Status was assessed at baseline and every 8 weeks. Follow-up occurred 4 weeks after the final dose, or every 4 weeks for the BSC arm.

### 4.7. Statistical Analysis

Data were analysed using SAS^®^ software version 9 (SAS Institute, Carry, NC, USA). All continuous variables were summarised using descriptive statistics. Categorical variables were reported using frequencies and percentages. Kaplan–Meier plots were used to visualise time-to-event data, and median value per treatment group calculated using the Kaplan–Meier method (95% CI calculated by the Brookmeyer–Crowley method). For the primary endpoint analysis, the DCR value and associated exact 95% CI per treatment group was presented. The Clopper–Pearson analysis method for the calculation of a CI for a single binomial proportion was used [33]. For each time-to-event endpoint, the survival distributions were compared between the treatment groups using a log-rank test with hazard ratio calculated using a proportional hazards model. All *p*-values quoted are one-sided. Conventional statistical significance was deemed to have been achieved when the one-sided *p*-value is <0.025. In this small early-phase study, *p*-values of ~0.1 or less were considered to provide a preliminary indication of efficacy.

## 5. Conclusions

The findings from this study suggest that CAP7.1 may offer a promising new therapeutic choice for BTC patients, who currently have very limited treatment options, and support its further clinical investigation.

## Figures and Tables

**Table 1:** Patient disposition.

Disposition	CAP7.1	BSC	Not Assigned Treatment	Total
All patients enrolled	14	13	1	28
Withdrawals before randomisation	NA	NA	1 *	1
Randomised	14	13	NA	27
Premature discontinuations	5	8	NA	13
Late screen failures/withdrawal of consent (i.e., randomised but not treated)	1 *	1 *	NA	2
Patients treated with BSC	NA	12	NA	12
Patients who crossed over from BSC to CAP7.1	NA	10	NA	10
All patients treated with CAP7.1	13	10	NA	23
Safety analysis set (SAS)	13	10	NA	23
Patients considered inappropriately randomised/eligibility violation	3 ^†^	1 *	NA	4
Lost-to-follow up/lack of post-randomisation data	0	1 *	NA	1
Full analysis set (FAS)	10	10	NA	20
Other significant protocol violations/lack of assessments	1 ^‡^	0	NA	1
Per-protocol analysis set (PAS)	9	10	NA	19

* These patients were excluded from all analysis sets; ^†^ These patients were not included in the FAS and PAS; ^‡^ This patient was not included in the PAS. BSC, best supportive care; NA, not applicable.

**Table 2:** Patient demographics and background characteristics.

Characteristic		CAP7.1	BSC
Number of patients		14	13
Mean age ± SD		60.3 ± 10.5	65.8 ± 8.70
Male, *n* (%)		8 (57)	6 (46)
Race Caucasian, *n* (%)		14 (100)	13 (100)
Histology, *n* (%)			
Differentiation			
	Well differentiated	1 (7)	2 (15)
	Moderately differentiated	7 (50)	5 (38)
	Poorly differentiated	2 (14)	5 (38)
	Undifferentiated	0	0
	Unknown	4 (29)	1 (8)
Type, *n* (%)			
	Distant metastatic	11 (79)	8 (62)
	Locally recurrent	4 (29)	6 (46)
TNM stage at diagnosis, *n* (%)			
	I	0	2 (15)
	II	2 (14)	4 (31)
	III	3 (21)	2 (15)
	IVA–IVB	9 (64)	5 (38)
	IVC	0	0
	I	0	2 (15)
Primary tumour site, *n* (%)			
	Intrahepatic		
	Extrahepatic (not further specified)	3 (21)	4 (31)
	Extrahepatic (Klatskin)	2 (14)	0
	Extrahepatic (Distal/ampulla of Vater)	4 (29)	1 (8)
	Gallbladder	2 (14)	2 (15)
	Multiple locations	2 (14)	1 (8)
	Cholangiocarcinoma not further specified	0	1 (8)
ECOG PS			
	0	4 (29)	9 (63)
	1	10 (71)	4 (31)
	2	0	0
	3	0	0
	4	0	0
Bilirubin (mg/dl)			
	mean ± SD	0.63 ± 0.71	0.58 ± 0.49
	median	0.4	0.4
Number of prior lines of chemotherapy, *n* (%)			
	1	9 (100)	6 (60)
	2	0	3 (30)
	3	0	0
	4	0	1 (10)
Previous surgery, *n* (%)			
	Resection	3 (21)	7 (50)
	Laparotomy	1 (7)	1 (8)

BSC, best supportive care; ECOG, Eastern Cooperative Oncology Group; PS, performance status; and SD, standard deviation. Bilirubin was assessed during the screening visit.

**Table 3:** Primary endpoint: disease control rate in the FAS and PAS patient populations.

Patients	FAS		PAS	
	CAP7.1 (*n* = 10)	BSC (*n* = 10)	CAP7.1 (*n* = 9)	BSC (*n* = 10)
Total number of patients with disease control	5	2	5	2
% of patients with disease control (95% CI) *	50.0 (18.7, 81.3)	20.0 (2.5, 55.6)	55.6 (21.2, 86.3)	20.0 (2.5, 55.6)
Treatment difference (95% CI)	30.0 (−18.44, 69.22)		35.56 (−12.80, 72.39)	
*p*-value for treatment difference ^†^	0.175		0.130	

* Using the Clopper–Pearson analysis method for the calculation of a confidence interval for a single binomial proportion; ^†^ 1-sided Fisher’s exact test; BSC, best supportive care; CI, confidence interval; FAS, full analysis set; and PAS, per-protocol analysis set.

**Table 4:** Adverse events (AEs) in the safety analysis set who received CAP7.1.

Adverse Events	CAP7.1 Randomised *n* = 13		BSC Before Crossover *n* = 10		BSC After Crossover *n* = 10		Total on CAP7.1 *n* = 23	
	*n* (%)	E	*n* (%)	E	*n* (%)	E	*n* (%)	E
Any AE	13 (100)	219	10 (100)	23	10 (100)	157	23 (100)	376
Any AE leading to discontinuation of study treatment	4 (31)	6	0	0	3 (30)	5	7 (30)	11
Any drug-related AE *	12 (92)	140	1 (10)	1	10 (100)	94	22 (96)	234
Any SAE	7 (54)	16	2 (20)	3	8 (80)	27	15 (65)	43
Any drug-related SAE *	6 (46)	8	0	0	5 (50)	13	11 (48)	21
Any fatal AE	4 (31)	6	0	0	4 (40)	5	8 (35)	11
Any drug-related fatal AE *	1 (8)	1	0	0	1 (10)	2	2 (9)	3

* ‘Related’ includes events whose relationship to study drug was rated as ‘Certain’, ‘Probable’, ‘Possible’, or ‘Missing’; E, number of events; and *n* (%), number (percentage) of patients with an event.
